# Protein Phosphatase 2A Mediates YAP Activation in Endothelial Cells Upon VEGF Stimulation and Matrix Stiffness

**DOI:** 10.3389/fcell.2021.675562

**Published:** 2021-05-13

**Authors:** Xiao Jiang, Jiandong Hu, Ziru Wu, Sarah Trusso Cafarello, Mario Di Matteo, Ying Shen, Xue Dong, Heike Adler, Massimiliano Mazzone, Carmen Ruiz de Almodovar, Xiaohong Wang

**Affiliations:** ^1^Laboratory of Molecular Ophthalmology, Department of Pharmacology, Tianjin Key Laboratory of Inflammation Biology, School of Basic Medical Sciences, Tianjin Medical University, Tianjin, China; ^2^Laboratory of Tumor Inflammation and Angiogenesis, Center for Cancer Biology, VIB, Leuven, Belgium; ^3^European Center for Angioscience, Medicine Faculty Mannheim, Heidelberg University, Mannheim, Germany; ^4^Department of Ophthalmology, Tianjin Medical University General Hospital, Tianjin, China; ^5^Key Laboratory of Immune Microenvironment and Disease, Ministry of Education, School of Basic Medical Sciences, Tianjin Medical University, Tianjin, China

**Keywords:** angiogenesis, YAP, PP2A, VEGF, matrix stiffness

## Abstract

Angiogenesis is an essential process during development. Abnormal angiogenesis also contributes to many disease conditions such as tumor and retinal diseases. Previous studies have established the Hippo signaling pathway effector Yes-associated protein (YAP) as a crucial regulator of angiogenesis. In ECs, activated YAP promotes endothelial cell proliferation, migration and sprouting. YAP activity is regulated by vascular endothelial growth factor (VEGF) and mechanical cues such as extracellular matrix (ECM) stiffness. However, it is unclear how VEGF or ECM stiffness signal to YAP, especially how dephosphorylation of YAP occurs in response to VEGF stimulus or ECM stiffening. Here, we show that protein phosphatase 2A (PP2A) is required for this process. Blocking PP2A activity abolishes VEGF or ECM stiffening mediated YAP activation. Systemic administration of a PP2A inhibitor suppresses YAP activity in blood vessels in developmental and pathological angiogenesis mouse models. Consistently, PP2A inhibitor also inhibits sprouting angiogenesis. Mechanistically, PP2A directly interacts with YAP, and this interaction requires proper cytoskeleton dynamics. These findings identify PP2A as a crucial mediator of YAP activation in ECs and hence as an important regulator of angiogenesis.

## Introduction

Extension of the vascular network is mediated by endothelial cell sprouting, migration, and proliferation. Recent studies have established Yes-associated protein (YAP), and its paralog transcriptional coactivator with PDZ-binding motif (TAZ; also known as WWTR1), as crucial regulators of angiogenesis (Kim et al., 2017; Wang et al., 2017). YAP/TAZ are the ultimate effectors of the Hippo signaling pathway. They shuttle between the cytoplasm and the nucleus, where they associate with transcriptional factor and regulate a set of target gene expression (Yu et al., 2012; Moya and Halder, 2019). The activity of YAP/TAZ can be suppressed by the core components of hippo pathway, containing a kinase cascade including MST1/2 and LATS1/2. MST1/2 phosphorylate LATS1/2, which then phosphorylate YAP/TAZ, causing their cytoplasmic sequestration and subsequent degradation (Totaro et al., 2018).

The activity of YAP/TAZ can be regulated by soluble growth factors and physical signals (Panciera et al., 2017). During angiogenesis, both vascular endothelial growth factor (VEGF) and extracellular matrix (ECM) stiffening activate YAP/TAZ in endothelial cells (Shen et al., 2020). VEGF stimulus or high stiffness reduce phosphorylation of YAP, which further lead to YAP activation and nuclear translocation (Wang et al., 2017; Meng et al., 2018). Interestingly, phosphorylation of MST1 did not change in response to VEGF stimulus, suggesting a potential MST-independent regulation of YAP activity by VEGF (He et al., 2018). This raises the question of whether YAP/TAZ regulation involves phosphatase activity.

Multiple protein phosphatases are identified as YAP phosphatase, including protein phosphatase 1 (PP1), protein phosphatase 2A (PP2A), and PTPN14 in different cell types (Schlegelmilch et al., 2011; Wang et al., 2011, 2012). Interestingly, in dominant negative PP2A expressing cells, VEGF induced ECs migration was significantly abolished (Urbich et al., 2002), suggesting that PP2A activity might be required for a VEGF stimulated angiogenic response. As an abundant cellular serine/threonine phosphatase, PP2A is involved in numerous signaling pathways (Janssens and Goris, 2001). In endothelial cells, PP2A is involved in regulating angiogenesis, vascular permeability, vascular remodeling in development and disease conditions (Le Guelte et al., 2012; Martin et al., 2013; Yun et al., 2019; Ehling et al., 2020). A recent study showed that in endothelial cells, YAP was a direct target of PP2A, and PP2A was involved in disturbed flow mediated YAP activation via Integrinα5β1 (Yun et al., 2019). Based on these evidences, in this study we wondered whether PP2A mediates YAP dephosphorylation in response to VEGF or high stiffness during angiogenesis.

Here, we found that PP2A is required for VEGF or high stiffness stimulated angiogenic responses. We demonstrate that the integrity of cytoskeleton is required for this regulation. *In vivo* pharmacological inhibition of PP2A activity reduced EC proliferation and inhibited angiogenesis in developmental and pathological angiogenesis models. These results provide further insight into the regulatory mechanism of YAP in ECs during angiogenesis.

## Results

### PP2A Regulates YAP Activity in ECs

**FIGURE 1:**
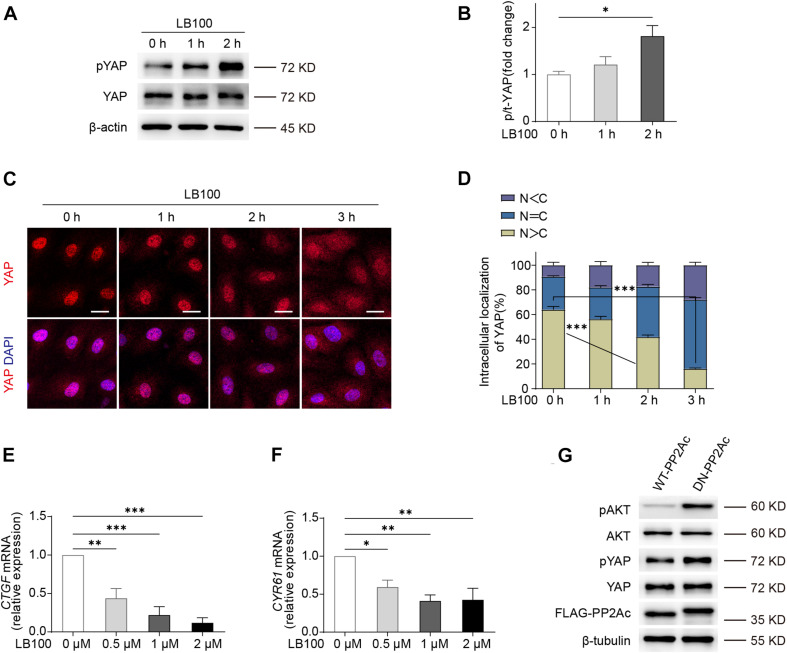
PP2A regulate YAP activity in ECs. **(A)** Western blot detection YAP phosphorylation status treated with 2 μM LB100 for the indicated time points. **(B)** Quantification of western blot shown in **(A)**. *n* = 4 independent experiments. **(C)** Representative images of HUVECs stimulated with 2 μM LB100 for the indicated time points and stained for YAP and DAPI. **(D)** Quantification of YAP subcellular localization treated as in **(C)**. Data from approximately 200 cells from 12 random fields of view (at least three independent experiments). N, nucleus; C, cytosol. N < C, nuclear staining was weaker than cytoplasm; N = C, nuclear staining was equal to cytoplasm; N > C, nuclear staining is stronger than cytoplasm. **(E,F)** qPCR analysis the expression of YAP target genes *CTGF, CYR61* in HUVEC treated with indicated dose of LB100 for 24 h. **(G)** Representative western blot showing pYAP status in HUVECs infected with WT-PP2Ac and DN-PP2Ac adenovirus for 36 h, pAkt was used as a positive control. Data are shown as mean ± SEM, one-way ANOVA followed by Tukey’s multiple comparisons test. ^∗^*p* < 0.05, ^∗∗^*p* < 0.01, ^∗∗∗^*p* < 0.001, ns indicates not significant. Scale bars, 20 μm.

We first determined whether PP2A regulates YAP activity in endothelial cells. For this, we used LB100, a pan-PP2A inhibitor that blocks the catalytic PP2A subunit (Hong et al., 2015). By checking the phosphorylation status of YAP, we found that LB100 treatment resulted in a hyper-phosphorylation of YAP in human umbilical vein endothelial cells (HUVECs) (Figures 1A,B). As the phosphorylation of YAP leads to cytoplasmic sequestration and inactivation, we further checked the subcellular localization of YAP. LB100 treatment time-dependently increased cytoplasmic localized YAP in HUVECs (Figures 1C,D). Analysis of mRNA levels of YAP/TAZ target genes *CTGF and CYR61* also showed a dose dependent decrease upon LB100 treatment (Figures 1E,F).

Next, we constructed an adenovirus vector expressing a dominant negative mutant form of the catalytic subunit C of PP2A, L199P (L199P and DN-PP2Ac), which is catalytically impaired (Evans et al., 1999). Wild-type (WT) PP2A catalytic C subunit (PP2Ac and WT-PP2Ac) were used as control. As Akt is known to be a PP2A substrate (Li et al., 2012; Tobisawa et al., 2017), we used phospho-Akt as a positive control. By infecting HUVECs with WT-PP2Ac or DN-PP2Ac adenovirus, we found that DN-PP2Ac indeed increased phospho-Akt (Figure 1G). Consistently, DN-PP2Ac also increased phospho-YAP, suggesting PP2A activity is required for YAP activation (Figure 1G).

### PP2A Activity Is Required for VEGF or High Stiffness Mediated YAP Activation

**FIGURE 2:**
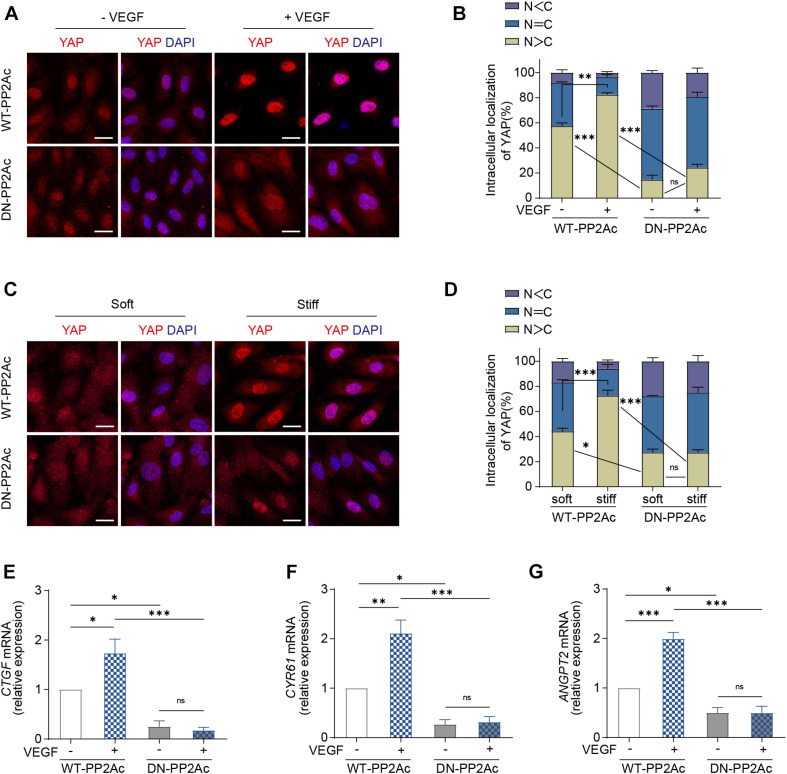
VEGF or high stiffness activation of YAP requires PP2A activity. **(A)** Representative images of YAP localization in HUVECs infected with WT-PP2Ac, DN-PP2Ac adenovirus for 24 h, starved in 2% FBS medium for another 12 h, and treated with or without 50 ng/mL VEGF for 3 h. **(B)** Quantification of YAP cellular localization for HUVECs treated as in **(A)**. Approximately 200 cells from 12 random fields of view from three independent experiments were quantified. N, nucleus; C, cytosol. **(C)** HUVECs were infected with WT-PP2Ac, DN-PP2Ac adenovirus for 36 h. The cells were then trypsinized and seeded on soft (0.2 kpa) and stiff (20 kpa) hydrogels. After 3 h, the cells were fixed and stained for YAP and DAPI. **(D)** Quantification of YAP cellular localization in HUVECs treated as in **(C)**. N, nucleus; C, cytosol. **(E–G)** Relative mRNA level of *CTGF* **(E)**, *CYR61* **(F)**, and *ANGPT2* **(G)** in HUVECs infected with WT-PP2Ac, DN-PP2Ac adenovirus, serum starved and stimulated with 50 ng/mL VEGF. Data are shown as mean ± SEM, one-way ANOVA followed by Tukey’s multiple comparisons test. ^∗^*p* < 0.05, ^∗∗^*p* < 0.01, ^∗∗∗^*p* < 0.001, ns indicates not significant. Scale bars, 20 μm.

Next, we aimed to understand whether PP2A is required for VEGF or high stiffness mediated YAP activation. For this, we used HUVECs transduced with WT-PP2Ac and DN-PP2Ac adenovirus and analyzed the subcellular localization of YAP. In WT-PP2Ac expressing cells, VEGF induced YAP nuclear translocation. However, in DN-PP2Ac expressing cells, VEGF failed to do so (Figures 2A,B). Similarly, high matrix stiffness increased nuclear localized YAP in WT-PP2Ac expressing cells, but not in DN-PP2Ac cells (Figures 2C,D). Additionally, DN-PP2Ac alone caused a significant reduction of nuclear localized YAP in all conditions (Figures 2A–D). Analysis of mRNA levels of *CTGF, CYR61*, and *ANGPT2* also revealed that YAP target genes upregulation induced by VEGF was only observed in WT-PP2Ac expressing ECs, but not in DN-PP2Ac ECs (Figures 2E–G). Again, with or without VEGF stimulation, DN-PP2Ac significantly reduced the gene expression of YAP target genes *CTGF, CYR61*, and *ANGPT2* compared with WT-PP2Ac (Figures 2E–G).

### VEGF or High Stiffness-Mediated EC Proliferation and Angiogenesis Requires PP2A Activity

**FIGURE 3:**
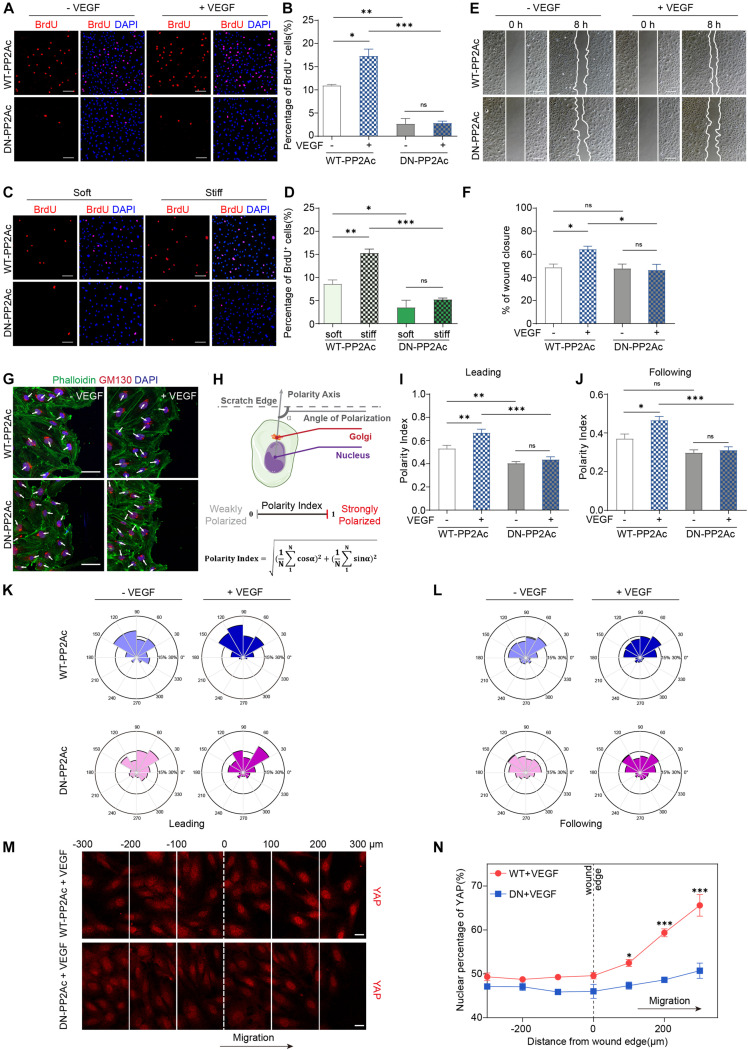
PP2A activity is required for EC proliferation and migration. **(A)** BrdU incorporation of HUVECs infected with WT-PP2Ac or DN-PP2Ac adenovirus, serum starved and stimulated with 50 ng/mL VEGF. **(B)** Quantification of BrdU^+^ cells of **(A)**. Data from 15 random fields of view (at least three independent experiments) were quantified. **(C)** HUVECs were infected with WT-PP2Ac orDN-PP2Ac adenovirus, trypsinized and seeded on soft (0.2 kpa) and stiff (20 kpa) hydrogels. After 6 h, the cells were fixed and stained for BrdU incorporation. **(D)** Quantification of BrdU^+^ cells of **(C)**. Cells from 12 random fields of view, from at least three independent experiments were quantified. **(E)** HUVECs were infected with WT-PP2Ac or DN-PP2Ac and the wound scratch assay was performed. Representative bright field images of the scratch assay showing HUVECs migration after 50 ng/mL VEGF treatment for 8 h. **(F)** Quantification of wound closure from **(E)**. Approximately 40 fields of view from five independent experiments were quantified. **(G)** Representative images of the wound scratch assay showing the polarity angles of WT- PP2Ac or DN-PP2Ac HUVECs after 50 ng/mL VEGF treatment for 8 h. GM130 labels Golgi apparatus. **(H)** Schematic of the Polarity Index calculation. Polarity axis of each cell was defined as the angle (α) between the scratch edge and the cell polarity axis (nucleus-to-Golgi apparatus vector). The polarity index was calculated according to the formula. **(I,J)** Polarity index of WT-PP2Ac or DN-PP2Ac leading cells **(I)** and following cells **(J)**. *n* = 100–120 leading cells and 320–350 following cells from three independent experiments. **(K,L)** Angular histograms showing the distribution of polarization angles of leading cells **(K)** and following cells **(L)**. *n* = 100–120 leading cells and 320–350 following cells from three independent experiments. **(M)** HUVECs were infected with WT-PP2Ac or DN-PP2Ac adenovirus for 30 h, afterward, the cells were starved for 12 h and a wound was made by scraping the confluent monolayer. 50 ng/mL VEGF was added in the medium. After 8 h, the cells were fixed and stained for YAP and DAPI. Representative immunofluorescent images of YAP localization were subdivided into 100 μm region of interests. The original wound edge was labeled as “0.” **(N)** Quantification of nuclear YAP fluorescence intensity of **(M)**. *n* = 7 fields from three independent experiments per condition. Data are shown as mean ± SEM, one-way ANOVA followed by Tukey’s multiple comparisons test in **(B,D,F,I,J)**, two-way ANOVA followed by Sidak multiple comparisons test in **(N)**. ^∗^*p* < 0.05, ^∗∗^*p* < 0.01, ^∗∗∗^*p* < 0.001, ns indicates not significant. Scale bars, 100 μm in **(A,C,E)** and 50 μm in **(G)**, 20 μm in **(M)**.

Yes-associated protein activity is required for multiple process during angiogenesis, including EC proliferation, migration and sprouting (Kim et al., 2017; Wang et al., 2017; Shen et al., 2020). To explore the biological consequences of PP2A mediated activation in response to VEGF or high stiffness, we performed a set of *in vitro* angiogenesis assays. To test whether PP2A has an effect on EC proliferation, we performed a BrdU incorporation assay in HUVECs infected by WT- or DN-PP2Ac expressing adenovirus. As expected, VEGF increased EC proliferation in WT-PP2Ac group, but had no obvious effect in DN-PP2Ac group (Figures 3A,B). To further understand the role of PP2A in stiffness regulated EC proliferation, we seeded HUVECs on polyacrylamide (PA) hydrogels with high or low stiffness. Similarly, while high stiffness increased proliferation in WT-PP2Ac expressing ECs, this effect was not observed in DN-PP2Ac expressing ECs (Figures 3C,D).

Expansion of the vasculature also requires ECs migration. *In vitro* scratch assay showed that while VEGF significantly induced EC migration and the closure of the gap in WT-PP2Ac expressing ECs, this effect was abolished in DN-PP2Ac ECs (Figures 3E,F). Consistently, DN-PP2Ac ECs failed to form *lamellipodia* after VEGF stimulation (Supplementary Figures 1A,B; Carvalho et al., 2019). Cell migration establishment also requires front-rear cell polarity. YAP/TAZ is required for cell polarity regulation, as depletion of YAP/TAZ impairs Golgi polarization in a wound scratch model (Mason et al., 2019). We therefore tested whether PP2A is also required for VEGF induced Golgi polarization. For this, we stained GM130 to label Golgi apparatus in the wound scratch model (Figure 3G), and quantified the polarity index of ECs (Figure 3H). VEGF increased significant Golgi polarization in both leading cells and following cells in WT-PP2Ac expressing ECs, but not in DN-PP2Ac ECs, suggesting that PP2A activity is also needed for cell polarity establishment (Figures 3I–L).

Yes-associated protein subcellular localization and activity are regulated by cell-cell interaction and cytoskeleton tension. In confluent endothelial monolayer, YAP nuclear enrichment increased preferentially in migrating cells beyond the wound edge in a wound scratch model (Mason et al., 2019), which contributes to the regulation of cell migration. However, in DN-PP2Ac expressing ECs, the nuclear enrichment of YAP in the wound edge was not observed, suggesting PP2A activity is required for YAP activation during cell migration (Figures 3M,N).

Cell geometry can be affected by matrix rigidity. High stiffness increases the spread cell area and elongation in ECs, and this morphological change could be regulated by YAP/TAZ (Dupont et al., 2011; Mason et al., 2019). By quantifying the spread cell area and elongation in WT-PP2Ac or DN-PP2Ac adenovirus infected ECs growing on soft or stiff matrix, we found that increasing the matrix stiffness significantly increased the cell spreading area and elongation in WT-PP2Ac ECs, however, this was not observed in DN-PP2Ac ECs (Supplementary Figures 1C–E). These data further suggest that PP2A is also involved in cell geometry change in response to matrix stiffness.

Yes-associated protein/TAZ is also crucial in regulating sprouting during angiogenesis (Wang et al., 2017). To evaluate the role of PP2A in vessel sprouting, we performed an *ex vivo* aortic ring assay. The quantification showed that inhibiting PP2A activity by LB100 significantly reduced vascularization and sprouting in the aortic ring model (Supplementary Figures 1F,G).

### PP2A Inhibition Impairs Developmental Angiogenesis *in vivo*

**FIGURE 4:**
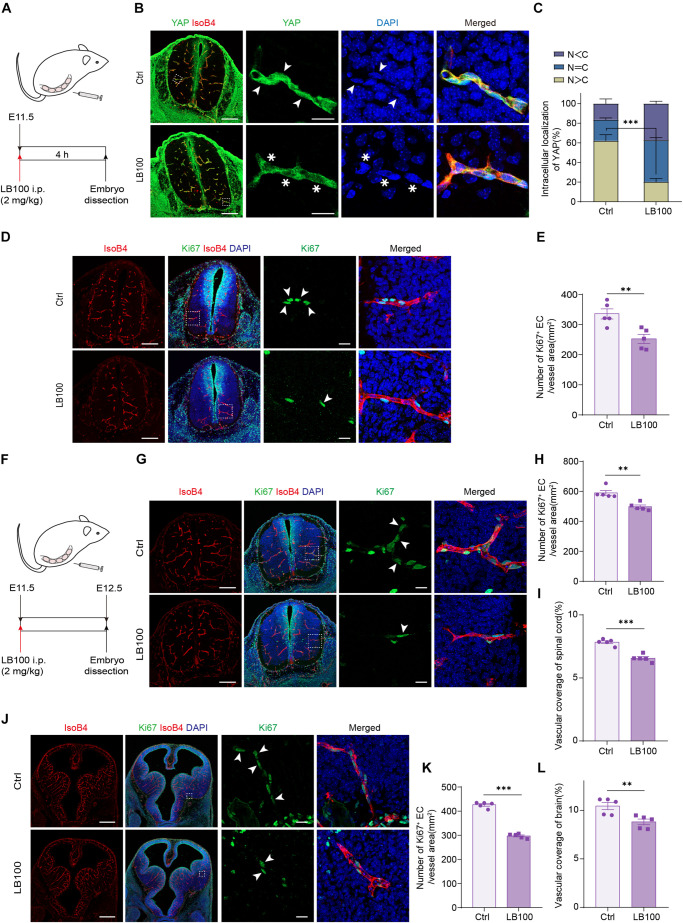
PP2A inhibition impairs developmental vascularization in embryos. **(A)** Scheme of LB100 injection in pregnant females. **(B)** Representative confocal images of spinal cord from mouse embryos treated as in **(A)** and stained for YAP, ECs (IsoB4), and nuclei (DAPI). Images are representative of 3 litters per condition. Arrowhead indicate nuclear YAP and asterisk indicate cytosolic YAP in ECs. **(C)** Quantification of YAP cellular localization in the spinal cord from embryos treated with vehicle or LB100 of **(B)**. N, nucleus; C, cytosol. *n* = 5 Vehicle and 5 LB100 embryos per condition. **(D)** Representative confocal images of spinal cord from embryos treated as in **(A)** and stained for Ki67, ECs (IsoB4), and nuclei (DAPI). Arrowhead indicate Ki67 positive ECs. Images are representative of 3 litters per condition. **(E)** Quantification of Ki67 positive ECs in the embryo spinal cord of **(D)**. *n* = 5 Vehicle and 5 LB100 embryos per condition. **(F)** Scheme of LB100 injection in pregnant females. **(G)** Representative confocal images of spinal cord from mouse embryos treated as in **(G)** and stained for Ki67, ECs (IsoB4), and nuclei (DAPI). Arrowhead indicate Ki67 positive ECs. Images are representative of 3 litters per condition. **(H)** Quantification of Ki67 positive ECs in the embryo spinal cord of **(H)**. *n* = 5 Vehicle and 5 LB100 embryos per condition. **(I)** Quantification of vascular coverage of the embryo spinal cord of **(H)**. *n* = 5 Vehicle and 5 LB100 embryos per condition. **(J)** Representative confocal images of brains from embryos treated as in **(G)** and stained for Ki67, ECs (IsoB4), and nuclei (DAPI). Arrowhead indicate Ki67 positive ECs. Images are representative of 3 litters per condition. **(K)** Quantification of Ki67 positive in brains of **(K)**. *n* = 5 Vehicle and 5 LB100 embryos per condition. **(L)** Quantification of vascular coverage ECs of **(K)**. *n* = 5 Vehicle and 5 LB100 embryos per condition. Data are shown as mean ± SEM, two tailed Student’s *t*-test. ^∗∗^*p* < 0.01 and ^∗∗∗^*p* < 0.001. Scale bars, 200 μm (lower magnification) and 20 μm (insets) in **(B,D,G)**, 500 μm (lower magnification) and 20 μm (insets) in **(J)**.

During embryonic mouse spinal cord development, VEGF expressed by the neuroepithelium activates YAP/TAZ in ECs and promotes vascularization (Wang et al., 2017). Thus, we explored whether PP2A activity is required for this process. For this, LB100 was administrated to pregnant females at embryonic day 11.5 (E11.5) and the embryos were dissected 4 h after injection (Figure 4A). Immunofluorescence showed that the nuclear localized YAP in ECs in LB100 treated group was significantly reduced (Figures 4B,C), suggesting that PP2A activity is required in ECs for YAP activation during developmental angiogenesis. Consistent with the inhibition of YAP activation, and the role of YAP in EC proliferation, we also detected reduced EC proliferation in spinal cords of LB100 (4 h) treated embryos (Figures 4D,E). We also dissected the embryos 24 h after LB100 injection (Figure 4F) and analyzed EC proliferation and vascularization. Consistently, LB100 treatment also reduced EC proliferation and blood vascular density in embryos 24 h after LB100 injection in pregnant females (Figures 4G,I). In addition, blood vessel density in the developing cortex also showed that LB100 treatment limited EC proliferation and impaired vascularization in another region of the central nervous system (Figures 4J–L).

### PP2A Inhibition Suppresses Pathological Retinal Angiogenesis in the Oxygen-Induced Retinopathy Model

**FIGURE 5:**
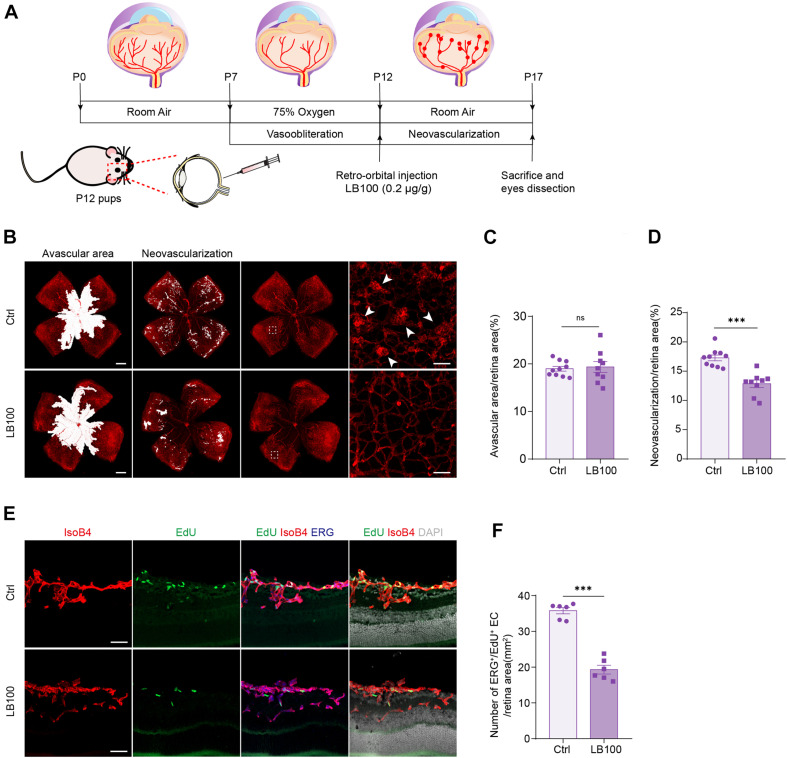
PP2A inhibition suppresses pathological retinal angiogenesis in the OIR model. **(A)** Schematic depiction of the mouse OIR model. Pups were placed in 75% oxygen from P7 to P12, and then returned to normal oxygen conditions. Vehicle (Ctrl) or LB100 (0.2 μg/g body weight) were injected retro-orbitally at P12. Pups were sacrificed and eyes were dissected at P17. **(B)** Representative confocal images of retina vasculature stained with IsoB4 in OIR retinas from vehicle (Ctrl) or LB100 treated pups. The avascular area, neovascularization area, and high magnification images for showing the neovascular tufts (indicated by arrowheads) are presented, respectively. **(C,D)** Avascular area and neovascularization quantification of **(B)**. *n* = 10 Vehicle and 9 LB100 eyes per condition. **(E)** Representative confocal images of retina sagittal sections costained with IsoB4, EdU (labels proliferating cells), and ERG (labels EC nuclei) in pups treated as in **(A)**. **(F)** Quantitative analysis of EdU^+^ ERG^+^ cell number of **(E)**. *n* = 6 Vehicle and 6 LB100 eyes per condition. Data are shown as mean ± SEM, two tailed Student’s *t*-test. ^∗∗∗^*p* < 0.001, ns indicates not significant. Scale bars, 500 μm (lower magnification) and 50 μm (insets) in **(B)**, 50 μm in **(E)**.

We next aimed to explore whether inhibiting PP2A activity is also necessary for pathological angiogenesis in the nervous system. For this, we deployed an oxygen-induced retinopathy (OIR) mouse model, which resembles human retinopathy of prematurity (ROP) and certain aspects of proliferative diabetic retinopathy (PDR) (Connor et al., 2009). For this, mouse pups were exposed to hyperoxia conditions (75% oxygen) from postnatal day 7 (P7) to P12, as previously described (Connor et al., 2009). Hyperoxia reduces the level of VEGF, which leads to vaso-obliteration. At P12, the pups were returned to ambient air, the relatively hypoxic environment led to high expression of VEGF, which in turn resulted in pathological neovascularization and formation of vascular tufts (Figure 5A). To test whether PP2A inhibition has an anti-angiogenic effect, which could be beneficial for preventing neovascularization, LB100 was retro-orbitally injected into the pups at P12. Saline vehicle injected in the same way served as control (Ctrl) (Figure 5A). Analysis of the avascular area revealed that LB100 did not significantly alter vaso-obliteration. However, by quantifying vascular tufts formation, we found that the neovascularization was significantly reduced in LB100 treated group (Figures 5B–D). We also analyzed whether EC proliferation was affected. We injected 5-ethynyl-2′-deoxyuridine (EdU) to label proliferating cells, and stained ERG to label the nucleus of endothelial cells. By quantifying the number of EdU^+^ ERG^+^ nuclei in the retina, we found that LB100 treatment significantly reduced the number of proliferating ECs (Figures 5E,F). As Angpt2 is a known YAP/target gene in endothelial cells (Choi et al., 2015; Wang et al., 2017; He et al., 2018), we determined the expression of *Angpt2* in the retinas by qPCR. The results showed that LB100 significantly reduced *Angpt2* expression, further proved an inhibitory effect of YAP by LB100 in the retinas (Supplementary Figure 2A).

The abnormal vascular growth in the OIR mice also results in impairment of vascular barrier, retinal hemorrhages and inflammation. Quantification of blood island showed that LB100 treatment significantly relieved retinal hemorrhages in the OIR model (Supplementary Figures 2B,C). Retinal inflammation also plays an important role in the pathogenesis of neovascular retinopathy. Consistently, the expression levels of monocyte chemotactic protein 1 (*Mcp-1*), intercellular adhesion molecule 1 (*Icam-1*), interleukin-1α (*Il-1a*), interleukin-1β (*Il-1b*), interleukin-6 (*Il-6*) and *Vegf* of the retina was significantly decreased after LB100 treatment (Supplementary Figures 2D–I). Collectively, these results indicate that PP2A inhibition suppresses YAP, limits EC proliferation, and protects vascular leakage in pathological angiogenesis.

### Cytoskeleton Dynamics Is Required for PP2A and YAP Interaction

**FIGURE 6:**
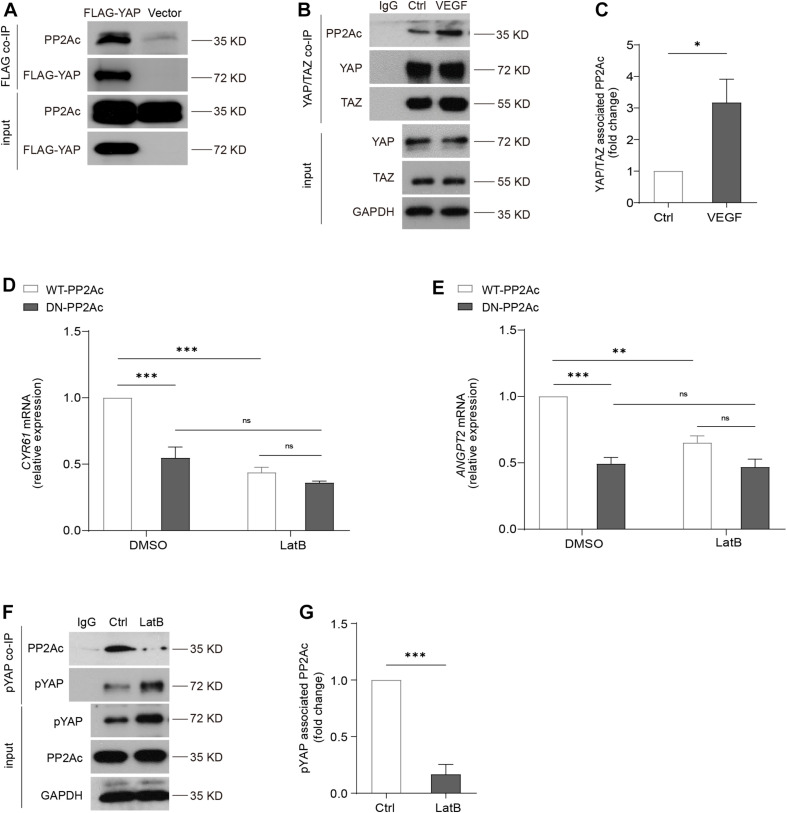
Cytoskeleton dynamics is required for PP2A and YAP interaction. **(A)** HEK293T cells were transfected with a control plasmid or a plasmid encoding Flag-YAP. Cell lysate were incubated with anti-Flag affinity beads and eluted with 3X-Flag peptide. Representative blots of this co-Immunoprecipitation (co-IP) showed PP2Ac as an interacting protein with Flag-YAP. **(B)** Representative blots of the co-Immunoprecipitation (co-IP) of endogenous YAP/TAZ and PP2Ac in HUVECs stimulated with 50 ng/mL VEGF for 2 h. **(C)** Quantification of co-IP shown in **(B)**. *n* = 3 independent experiments. **(D,E)** HUVECs were infected with WT-PP2Ac or DN-PP2Ac adenovirus for 36 h, followed by LatB (0.05 μg/mL) treatment for 6 h. The expression of YAP target genes *CYR61 and ANGPT2* were analyzed by qPCR. **(F)** Representative blots of the co-Immunoprecipitation (co-IP) of pYAP and PP2Ac in HUVECs treated with LatB for 2 h. **(G)** Quantification of co-IP shown in **(F)**. *n* = 3 independent experiments. Data are shown as mean ± SEM, one-way ANOVA followed by Tukey’s multiple comparisons test in **(D,E)**, two tailed Student’s *t*-test in **(C,G)**. ^∗^*p* < 0.05, ^∗∗^*p* < 0.01, ^∗∗∗^*p* < 0.001, ns indicates not significant.

We next aimed to investigate how PP2A is involved in the regulation of YAP activation. It was shown that PP2Ac directly bind to YAP, and this interaction is associated with cell density or presence of α-catenin in a human epidermal keratinocyte line (Schlegelmilch et al., 2011). In HEK293T cells, we transfected Flag-YAP or the control vector plasmid (vector). YAP, and its bounded protein patterns, were then immunoprecipitated by using anti-Flag M2 magnetic beads. Western blot showed that PP2Ac binds to Flag-YAP and confirmed a physical interaction of PP2Ac and YAP (Figure 6A). In HUVECs, we also performed co-IP for YAP/TAZ. Consistently, in ECs PP2Ac also was found associated with YAP/TAZ and this interaction was further enhanced by VEGF stimulation (Figures 6B,C). A previous study (He et al., 2018) showed that VEGF-mediated activation of YAP is independent of MST kinase, and our own data also showed that the binding of MST and PP2Ac was not changed by VEGF treatment (Supplementary Figure 2J). Altogether suggesting that an interaction between phosphatase PP2A and YAP is required YAP dephosphorylation and activation in response to VEGF stimulation.

By using an actin-disrupting agent, Latrunculin B (LatB) to interrupt actin polymerization in ECs, YAP/TAZ activity could not be activated by VEGF (Wang et al., 2017), suggesting YAP/TAZ activation requires an intact actin cytoskeleton. DN-PP2Ac suppressed expression of YAP target genes including *CYR61* and *ANGPT2*, compared with WT-PP2Ac cells. Interestingly, in LatB treated ECs, no significant difference was observed between DN-PP2Ac and WT-PP2Ac expressing ECs, suggesting the regulation of PP2A on YAP requires actin cytoskeleton (Figures 6D,E). Next, we questioned whether LatB could affect the binding of PP2Ac to YAP. For this, we again performed co-IP experiment. The result showed that while phospho-YAP was increased upon LatB treatment, the binding of PP2Ac and pYAP was strongly reduced (Figures 6F,G). The above results further suggested that PP2A regulation of YAP requires cytoskeleton dynamics.

## Discussion

**FIGURE 7:**
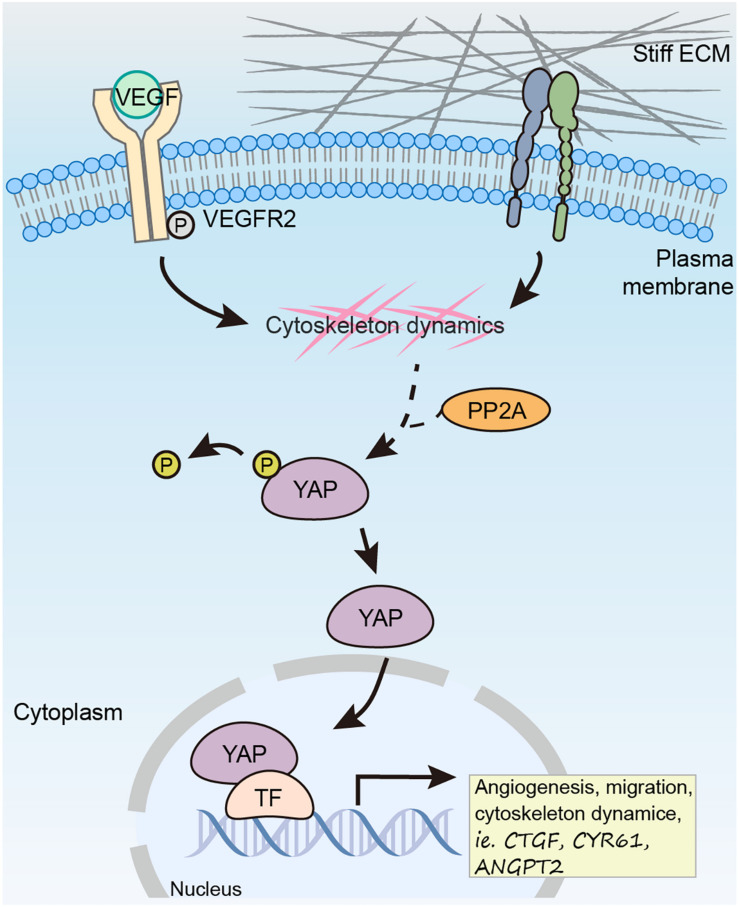
Working model showing PP2A mediates dephosphorylation of YAP in response to VEGF and high stiffness to promote angiogenesis in ECs.

Proper control of angiogenesis is crucial for organ vascularization and growth. Recent studies have defined YAP/TAZ as essential factor in regulating angiogenesis. In response to pro-angiogenic stimuli such as VEGF or ECM rigidity, the phosphorylation of YAP/TAZ is reduced, which further leads to its nuclear translocation and activation. Although there are mounting evidences showing the important role of YAP/TAZ in angiogenesis in multiple model organisms, it is not clear why the pro-angiogenic stimulus result in hypo-phosphorylation of YAP/TAZ, as the canonical Hippo signaling upstream kinase MST is not involved in this regulation (He et al., 2018). In this study, we prove that PP2A mediates hypo-phosphorylation of YAP in ECs in response to pro-angiogenic stimulus, specifically, VEGF and high stiffness (Figure 7).

Protein phosphatase 2A is one of the major Ser/Thr phosphatases that regulates different biological processes such as cell proliferation, apoptosis and signaling transduction by dephosphorylating many critical molecular such as Akt, cMyc, P53, β-catenin (Seshacharyulu et al., 2013). Aberrant expression of PP2A is associated with various human malignancies, as loss of its phosphatase activity has been found in several type of tumors (Janssens et al., 2005; Mumby, 2007). In blood vessels, it has been reported that PP2A is involved in regulating multiple steps of angiogenesis, including endothelial tube formation, lumen stabilization, vascular remodeling, and vascular permeability (Le Guelte et al., 2012; Martin et al., 2013; Ehling et al., 2020). During atherogenesis, PP2A is also involved in inflammatory activation of the endothelium (Yun et al., 2016, 2019). These studies highlight the importance of PP2A in regulating blood vessel formation and homeostasis.

Protein phosphatase 2A holoenzyme consists a core enzyme composed of the structural A and catalytic C subunits, and regulatory B subunit (Schuhmacher et al., 2019). There are 2 A isoforms, 2 C isoforms, and four families of B subunit, each containing several isoforms, which mediate selective substrate binding and catalytic activity (Schuhmacher et al., 2019). Thus, the modulation of PP2A activity by different B subunit highlights the complexity. In endothelial cells, the important role of PP2A-B55α subunit have been recognized. Two studies have shown that B55α plays a crucial role in vessel stabilization in both zebrafish and mouse (Martin et al., 2013; Ehling et al., 2020). During mouse embryonic development, endothelial specific knockout of B55α leads to embryonic lethality, suggesting an essential role of B55α in the developing vasculature (Ehling et al., 2020). Importantly, another study also reported B55α subunit recruit PP2A to phosphodiesterase 4D5 (PDE4D5), and such interaction stabilize B55α-PP2A complex and then dephosphorylate and activate YAP. Due to the important role of B55α in ECs and its interaction with YAP, although in this study we did not focus on identifying the varying regulatory B subunit, it is tempting to speculate that B55α might be involved in this regulation.

The role of PP2A in regulating Hippo signaling pathway was reported by several studies. The canonical Hippo pathway consists of a core kinase cascade in which MST phosphorylates LATS, which further phosphorylates YAP/TAZ. In the MST-LATS-YAP/TAZ signaling axis, studies have shown that PP2A acts as a phosphatase and could dephosphorylate MST and YAP. Regulation of MST by PP2A has been observed in *Drosophila* and mammals, where PP2A directly interact with MST and dephosphorylate MST (Ribeiro et al., 2010; Couzens et al., 2013; Chen et al., 2019). Furthermore, PP2A inhibition reactivates Hippo signaling via targeting MST, which has anti-tumor effects (Tang et al., 2020). PP2A also interact with YAP to induce dephosphorylation of YAP, thereby disrupting the binding to 14-3-3 and leads to YAP activation (Schlegelmilch et al., 2011; Wang et al., 2011; Yun et al., 2019). However, PP1, but not PP2A, is responsible for dephosphorylation of TAZ (Liu et al., 2011). Thus, in this study, we mainly focused on investigating YAP. However, PP2A inhibitor LB100 or dominant negative form of PP2A treatment might also lead to TAZ activation via an upstream regulation of MST-LATS-TAZ axis. Interestingly, Akt, which is a known substrate of PP2A (Li et al., 2012; Tobisawa et al., 2017), could also be a regulator of YAP (Basu et al., 2003; Strano et al., 2005). It was reported that Akt could phosphorylate YAP, and such modification impairs YAP nuclear translocation and cotranscriptional activity (Basu et al., 2003; Strano et al., 2005). Thus, it would be interestingly to test whether a PP2A-Akt-YAP regulatory axis exists in ECs, as Akt is known to be a crucial regulator for EC migration and survival (Dimmeler and Zeiher, 2000; Lamalice et al., 2007).

ECs are influenced by the mechanical properties of the environment. Stiff matrix promotes EC proliferation and can lead to defects of vascular integrity (Huynh et al., 2011; Yeh et al., 2012). YAP/TAZ works as mechanotransducers to regulate cell behaviors. Various mechanical cues, such as ECM rigidity, shear stress and stretching regulate YAP/TAZ activity (Dupont et al., 2011; Benham-Pyle et al., 2015; Wang K.C. et al., 2016; Wang L. et al., 2016; Chang et al., 2018; Li et al., 2019). In the process of pathological angiogenesis in highly fibrotic solid tumor tissue, both VEGF and matrix stiffness trigger YAP/TAZ activation and lead to abnormal vessel growth and impaired vessel integrity (Shen et al., 2020). As we identified PP2A as a crucial mediator during this regulation, it might be valuable to consider PP2A as a target also for controlling solid tumor angiogenesis. Indeed, it was shown that LB100 treatment reduced tumor burden and tumor vascular density in a LLC lung cancer model (Ehling et al., 2020). Notably, LB100 as the pan-PP2A inhibitor, was approved for phase I clinical study in patients. There are also two phase II clinical studies on going (Chung et al., 2017)^1^. This further highlights the potential of PP2A inhibitors as an anti-pathological angiogenesis strategy.

In summary, in this study we show that PP2A mediates hypo-phosphorylation of YAP in ECs in response to VEGF or high stiffness. PP2A inhibition diminished VEGF or high stiffness triggered EC proliferation and angiogenesis. In a pathological angiogenesis model, blocking PP2A reduced abnormal vessel growth and inhibited vascular leakage. Thus, it provide new insight for considering the PP2A-YAP axis for therapeutic interventions in angiogenesis disorders.

## Materials and Methods

### Animals

All study protocols involving the use of animals were approved by the Institutional Animal Care and Use Committee of Tianjin Medical University. Adult wild-type C57BL/6J and neonatal C57BL/6J mice with mothers were purchased from Model Animal Research Center of Nanjing University.

### Cell Culture

Human umbilical vein endothelial cells were cultured with M199 (Gibco^®^, Life Technologies, United States) supplemented with 20% FBS, 100 U/mL penicillin and 100 μg/mL streptomycin (HyClone^TM^, GE Healthcare Life Sciences, United States), glutamine, heparin, thymidine and endothelial cell growth factors (ECGF, Sigma-Aldrich, United States) as previously described (Fu et al., 2011) and were used from passage 2 to passage 5. For stimulation experiments, cells were starved overnight in ECGF free M199 medium supplemented with 2% FBS. HEK293T cells were cultured in Dulbecco Modified Eagle’s Medium containing 10% FBS penicillin (100 U/mL) and streptomycin (100 μg/mL).

### Antibodies and Reagents

Rabbit monoclonal antibody (mAb) against YAP (Cat No. 52771), rabbit mAb against Ki67 (Cat No. 15580), mouse mAb against human CD31 (Cat No. 9498), rabbit mAb against ERG (Cat No. 92513) and rat mAb against BrdU (Cat No. 6326) were purchased from Abcam (Cambridge, United Kingdom). Rabbit mAb against YAP (Cat No. 14074), Rabbit mAb against pYAP (Cat No. 4911) and rabbit mAb against MST1 (Cat No. 14946) were from Cell Signaling Technology (Boston, MA, United States). Mouse mAb against β-actin, Mouse mAb against GAPDH and Mouse mAb against β-tubulin were from Utibody (Tianjin, China). LB100 (Cat No. s7537) was from Selleck (Houston, TX, United States). LatB (Cat No. L5288) was from Sigma Aldrich (St. Louis, MO, United States). Recombinant Human VEGF (Cat No. 293-VE) was from R&D systems (Minneapolis, MN, United States).

### PP2A Recombinant Adenovirus Construction and Infection

Generation of L199P (DN-PP2Ac) mutants was as previously described (with point mutations of leu 199 to pro). Adenovirus expressing green fluorescent protein (Ad-GFP), Ad-Flag-tagged human WT-PP2Ac and DN-PP2Ac mutants were from GeneChem (Shanghai, China). HUVECs were infected with adenovirus at multiplicity of infection (MOI) ≈100. A total of 36–48 h after infection, cells were used for analysis.

### Mouse Embryos Processing

To analyze the vasculature of mouse embryos, LB100 was dissolved in saline solution (0.9% NaCl) and injected intraperitoneally with a concentration of 2 mg/kg body weight at E11.5. Embryos were then dissected at indicated time points and fixed by 4% paraformaldehyde/PBS at 4°C overnight. Afterward, they were transferred to 30% sucrose/PBS at 4°C overnight and subsequently embedded in optimal cutting temperature compound (OCT) (Sakura, Japan) and frozen at −80°C. Serial 20 μm (for spinal cord) and 60 μm (for brain) -thick sections were cut using a cryostat (CM1950, Leica, Germany).

### Immunofluorescence

Cryosections were washed, permeabilized in PBS containing 0.3% TritonX-100 then blocked in PBS solution with 2% BSA, 0.3% TritonX-100 for 1 h, and incubated with primary antibodies (YAP, 1:200; ki67, 1:1000) at 4°C overnight. After washing, the cryosections were incubated with corresponding secondary antibodies for 2 h at RT. Images were collected on a confocal fluorescence microscope (LSM 800, Carl Zeiss, Germany). At least four images from each tissue were used for analyze.

### Oxygen-Induced Retinopathy Model

Oxygen-induced retinopathy model was performed as previously described (Connor et al., 2009; Dong et al., 2021). Briefly, neonatal C57BL/6J mice with the nursing mother at day P7 were exposed to hyperoxia (75% O_2_) for 5 days and then returned to room air at P12. For applying treatment, LB100 was dissolved in saline solution (0.9% NaCl) and injected retro-orbitally with a concentration of 0.2 μg/g body weight at P12. The retinas were collected at P17 for analysis.

### Retina Dissection, Processing, and Staining

Mice were sacrificed and eyes were enucleated. The eyes were fixed in 4% PFA/PBS for 4 h at 4°C. Retinas were dissected, washed with PBS and permeabilized with PBS containing 1% TritonX-100 overnight at 4°C then blocked in PBS containing 5% BSA, 0.5% TritonX-100. After blocking, for visualization of retinal vasculature in OIR models, flat-mounted retinas were stained with isolectinGS-IB4 (Alexa Fluor 568-conjugated, 1:100, I21413, Invitrogen, United States) for 2 h at RT. Flat-mounted retinas were analyzed using a confocal fluorescence microscope (LSM 800, Carl Zeiss, Germany).

### Morphometric Analyses

Morphometric analyses of the retinas were performed using ImageJ software and Adobe Photoshop software. To determine the amount of regression and neovascularization, the number of pixels in the avascular regression area and neovascular area was measured using the Lasso tool of Adobe Photoshop software as described (Connor et al., 2009), and divided by the number of pixels in the total retinal area and presented as a percentage.

### EdU Injection in Pups

A total of 50 μg/g body weight EdU (E6032, US EVERBRIGHT INC., China) was i.p. injected into pups at P17 in OIR model 2.5 h before sacrifice. Eyes were enucleated and fixed in 4% PFA for 1 h at 4°C. Afterward, the eyes were transferred to 30% sucrose/PBS at 4°C overnight and embedded in OCT (Sakura, Japan) and stored at −80°C. Sagittal cryosections of the eyes were made for analysis. EdU^+^ cells in the cryosections of retinas were detected by using YF^®^ 488 Click-iT EdU Stain Kits (C6033, US EVERBRIGHT INC., China), according to the manufacturer’s instructions. ECs were counterstained with ERG (1:400) and isolectinGS-IB4 (1:200). The numbers of EdU^+^ERG^+^ cells were counted from six sagittal eye sections. To ensure that similar areas were used for doing quantification for each eye, we always use sections intersect the optic nerve area to maintain consistency.

### Mouse Aortic Ring Assay

The aortic ring assay was performed as previously described (Baker et al., 2011). Briefly, the thoracic aorta was sectioned into 1-mm long aortic rings and cultured in Opti-MEM^TM^ (Thermo Scientific, US) with 100 U/mL penicillin and 100 μg/mL streptomycin overnight. Aortic rings were encapsulated in growth factor reduced Matrigel (Corning, United States) in 24-well plates. The aortic ring was then cultured in Opti-MEM^TM^ supplemented with 2.5% FBS, 30 ng/mL VEGF with or without LB100 (4 μM) in a humidified 37°C, 5% CO_2_ incubator for 5 days. Images were acquired by using a Nikon TI2-U microscope. Images were analyzed with NIH ImageJ software.

### RNA Extraction and Quantitative Real-Time PCR Analysis

For animals, eyes were enucleated from pups at P17 in OIR model. Retinas were dissected and homogenized in TRIzol^®^ reagent (Invitrogen, United States). For *in vitro* cultured cells, the cells were harvested for RNA extraction using TRIzol^®^ reagent. RNA samples were reverse-transcribed to complementary DNA (cDNA) using TransScript One-Step gDNA Removal and cDNA Synthesis SuperMix (TransGen, China). qPCR was performed using *PerfectStart*^TM^ Green qPCR SuperMix (TransGen, China) and was processed with QuantStudio 5 Real-Time PCR system (Applied Biosystems, United States). GAPDH was used as internal control. All qPCR results were obtained from at least three biological repeats.

### Immunoprecipitation and Immunoblotting

Co-IP assays were performed as previously described (Wang et al., 2017). Cells were lysed using mild lysis buffer [20 mM Tris at pH 7.5, 150 mM NaCl, 5 mM EDTA, 1% NP-40, 10% Glycerol, 1X protease inhibitor cocktail, and 1X phosphatase inhibitor (Roche)]. Cell lysates were centrifuged for 15 min, and supernatants were used for immunoprecipitation. Anti-YAP/TAZ antibody or anti-pYAP antibody (Cell Signaling Technology, United States) was incubated with the supernatant overnight on a rotor, and protein A/G–magnetic beads (Thermo Scientific, United States) were added in for 2 more hours. Immunoprecipitates were washed and proteins were eluted with SDS sample buffer. For analyzing the interaction of YAP and PP2Ac in HEK293T, the cells were transfected with Flag-tagged human YAP (Flag-YAP). Empty vector was used as control. After 36 h, cell lysate was collected and incubated with anti-Flag M2 magnetic beads (Sigma-Aldrich) to capture the Flag protein complex. After binding, the magnetic beads were washed, 3X-Flag peptide was applied to elute the Flag-protein complex.

### Immunofluorescence of Cells in Culture

For YAP staining, HUVECs were cultured in collagen coated coverslips in 24-well plates or on PA hydrogels. After treatment, cells were fixed with 4% PFA/PBS for 15 min at RT. Afterward, cells were permeabilized with 0.2% Triton X-100 PBS, blocked in 2% BSA, 0.2% Triton X-100 PBS, and incubated with primary antibodies (YAP, 1:200; GM130, 1:50; human CD31, 1:100). After washing, cells were incubated with secondary antibodies. Phalloidin (1:100, CA1610, Solarbio, China) was used together with secondary antibodies. Images were acquired by confocal microscope (LSM 800, Carl Zeiss, Germany). More than 8 fields of view from at least three independent experiments were randomly chosen. Cells presenting preferential nuclear YAP localization, equal nuclear or cytoplasmic, or mainly cytoplasmic YAP localization were counted blindly.

### Polyacrylamide Gel Manufacturing

Polyacrylamide gels were prepared as previously described (Yeh et al., 2012). Briefly, solutions were prepared by using acrylamide (40% w/v solution; Sigma-Aldrich, Germany; A4058) and bis-acrylamide crosslinker (2% w/v solution, Sigma-Aldrich, Germany; 111-26-9). Gels with different stiffness were prepared by varying the final concentrations of acrylamide and bis-acrylamide. To remove oxygen from the solutions, the mixtures were degassed for 15 min. To polymerize the mixtures, 30 μL of 10% w/v ammonium persulfate (Bio-Rad, United States; 1610700) and 20 μL of N,N,N9,N9-tetramethylethylenediamine (TEMED; Bio-Rad, United States; 1610800) were added to yield a final volume of 5 mL. Gel surfaces were then activated by exposing the heterobifunctional crosslinker Sulfo-SANPAH (Pierce, United States; 22589) at 0.5 mg/mL in 50 nM HEPES (Sigma-Aldrich, Germany; H3375) to UV light. After activation, the gels were washed with PBS to remove excess crosslinker. Gels were then coated with 0.1 mg/mL collagen (Sigma-Aldrich, Germany; F0895) at 37°C for 1 h or overnight at 4°C.

### BrdU Incorporation in ECs

The BrdU incorporation assay was performed as previously described (Shen et al., 2020; Dong et al., 2021). Briefly, HUVECs were cultured in collagen coated coverslips in 24-well plates or on PA hydrogels. After indicated treatment, BrdU (10 μM) was added and incubation of the cultures continued for 4 h at 37°C. Cells were fixed in 4% PFA/PBS for 20 min and blocked in PBS containing 2% BSA, 0.3% TritonX-100 for 30 min at RT. Unmasking was done by adding ice-cold 0.1 M HCl for 20 min followed by 2 M HCl for 30 min. Neutralization was done prior to primary antibody incubation with sodium borate buffer (0.1 M Na_2_B_4_O7 in water, PH8.5) for 15 min. An anti-BrdU antibody (1:250) was incubated in blocking solution overnight at 4°C and corresponding secondary antibody was incubated for 2 h at RT. Nuclei were labeled with DAPI (1:1000, D1306, Invitrogen, United States). Images were obtained using a fluorescence microscope (Ti2-U, Nikon, Japan). Quantification was done blind to the experimental condition.

### Scratch Assay

To analyze the EC migration, scratch assay was performed as previously described (Tisch et al., 2019). HUVECs were plated on 6-well plates and infected with adenoviruses for 30 h. After overnight starvation, a wound was made by scraping the cell monolayer with a 200 μL pipette tip, and cells were stimulated with or without VEGF (50 ng/mL). Pictures were acquired at time-point zero and 8 h after incubation at 37°C. The percentage of wound closure between 0 and 8 h was analyzed with ImageJ software. Results are from five independent experiments, for each treatment eight fields of view were analyzed.

### Polarity Index Calculation

Cell polarity analysis was performed as previously described (Dubrac et al., 2016; Carvalho et al., 2019). Briefly, HUVECs were plated on coverslips and infected with adenoviruses for 30 h. After overnight starvation, a wound was made by scraping the cell monolayer with a 200 μL pipette tip, and cells were stimulated with or without VEGF (50 ng/mL). After 8 h, the culture was fixed and stained with GM130 (Alexa Fluor^®^ 555-conjugated, 1:50, 560066, BD, United States), Phalloidin (1:100, CA1620, Solarbio, China) and DAPI (1:1000). Images were obtained by confocal microscope (LSM 900, Carl Zeiss, Germany) and analyzed by ImageJ software. Leader cells were identified as the first row of cell directly in contact with the scratch, the follower cells comprising the second to third rows of cells away from the scratch. The polarity of each cell was defined as the angle (α) between the Golgi-nuclei axis and the scratch line as shown in Figure 3H. The polarity index was calculated following the formula below.

$$\mathrm{Polarity}\mathrm{Index}=\sqrt{(\frac{1}{\text{N}}\sum_{1}^{\text{N}}\cos^{2}{\left(\frac{1}{\text{N}}\sum_{1}^{\text{N}}\text{sin}\right)}^{2}}$$

## Statistical Analysis

Results were expressed as the mean ± SEM. To calculate statistical significance, the student’s *t*-test (two-tailed), one-way ANOVA followed by Tukey’s multiple comparisons test or two-way ANOVA followed by Sidak multiple comparisons test were used. *p*-value < 0.05 was considered significant. All calculations were performed using Prism software.

## Data Availability Statement

The original contributions presented in the study are included in the article/Supplementary Material, further inquiries can be directed to the corresponding author/s.

## Ethics Statement

The animal study was reviewed and approved by the Institutional Animal Care and Use Committee of Tianjin Medical University.

## Author Contributions

CR and XW designed the project. XJ, JH, ZW, SC, MD, YS, XD, HA, and XW carried out the experiments. XJ, JH, ZW, and XW analyzed the data. MM, CR and XW supervised the study. XJ, CR, and XW wrote the manuscript with the input from all authors.

## Conflict of Interest

The authors declare that the research was conducted in the absence of any commercial or financial relationships that could be construed as a potential conflict of interest.

## Footnotes

1 https://clinicaltrials.gov/ct2/results?cond=&term=LB100&cntry=&state=&city=&dist

## Supplementary Material

Supplementary Figure 1 PP2A activity is required for EC cytoskeleton change and sprouting. **(A)** Representative images of HUVECs stained with Phalloidin (actin) showing that *lamellipodia* formation upon VEGF (50 ng/mL for 30 min) treatment in WT-PP2Ac or DN-PP2Ac expressing HUVECs. **(B)** Quantification of the number of HUVECs with *lamellipodia* of **(A)**. Cells in nine random fields of view (≈120 cells) were quantified. **(C)** Representative images of WT-PP2Ac or DN–PP2Ac expressing HUVECs seeded on soft (0.2 kpa) and stiff (20 kpa) hydrogels stained for Phalloidin (actin). **(D,E)** Quantification of individual cell area and circularity in **(C)**. A, area; P, perimeter. Cells in 10 random fields of view (≈50 cells per condition) were quantified. **(F)** Representative images of mice aortic rings treated with vehicle (Ctrl) or LB100 (4 μM). **(G)** Quantification of microvascular sprouting area of **(F)**. (*n* = 3–5 aortic ring per condition.) Data are shown as mean ± SEM, one-way ANOVA followed by Tukey’s multiple comparisons test in **(B,D,E)**, two tailed Student’s *t*-test in G. ^∗^*p* < 0.05, ^∗∗^*p* < 0.01, and ^∗∗∗^*p* < 0.001, ns indicates not significant. Scale bars, 50 μm (lower magnification) and 20 μm (insets) in **(A)**, 20 μm in **(C)**, 500 μm in **(F)**.

Supplementary Figure 2 PP2A inhibition reduces vascular leakage in the OIR model. **(A)** qPCR analysis of the expression of YAP target gene *Angpt2* in retinas of vehicle or LB100 treated OIR pups. (*n* = 5 Vehicle and 9 LB100 mice per condition.) **(B)** Representative images of the outer surface of the OIR retinal cup at P17. Blood island formation indicates hemorrhages in the retinas. **(C)** Quantification of blood island area out of the total retinal area of **(B)**. (*n* = 5 Vehicle and 7 LB100 mice per condition). **(D–I)** qPCR analysis of the expression of *Mcp-1, Icam-1, Il-1a, Il-1b, Il-6*, and *Vegf* in retinas treated as in **(A)**. (*n* = 5 Vehicle and 9 LB100 mice per condition.) **(J)** Representative blots of the co-Immunoprecipitation (co-IP) of endogenous MST1 and PP2Ac in HUVECs stimulated with 50 ng/mL VEGF for 2 h. Data are shown as mean ± SEM, two tailed Student’s *t*-test. ^∗^*p* < 0.05, ^∗∗^*p* < 0.01, and ^∗∗∗^*p* < 0.001. Scale bars, 1 mm.
