# Supplementary figures and images for: Protein Phosphatase 2A Mediates YAP Activation in Endothelial Cells Upon VEGF Stimulation and Matrix Stiffness
Source: Front Cell Dev Biol. 2021 May 13;9:675562. doi: 10.3389/fcell.2021.675562 (PMC8158299; doi:10.3389/fcell.2021.675562)

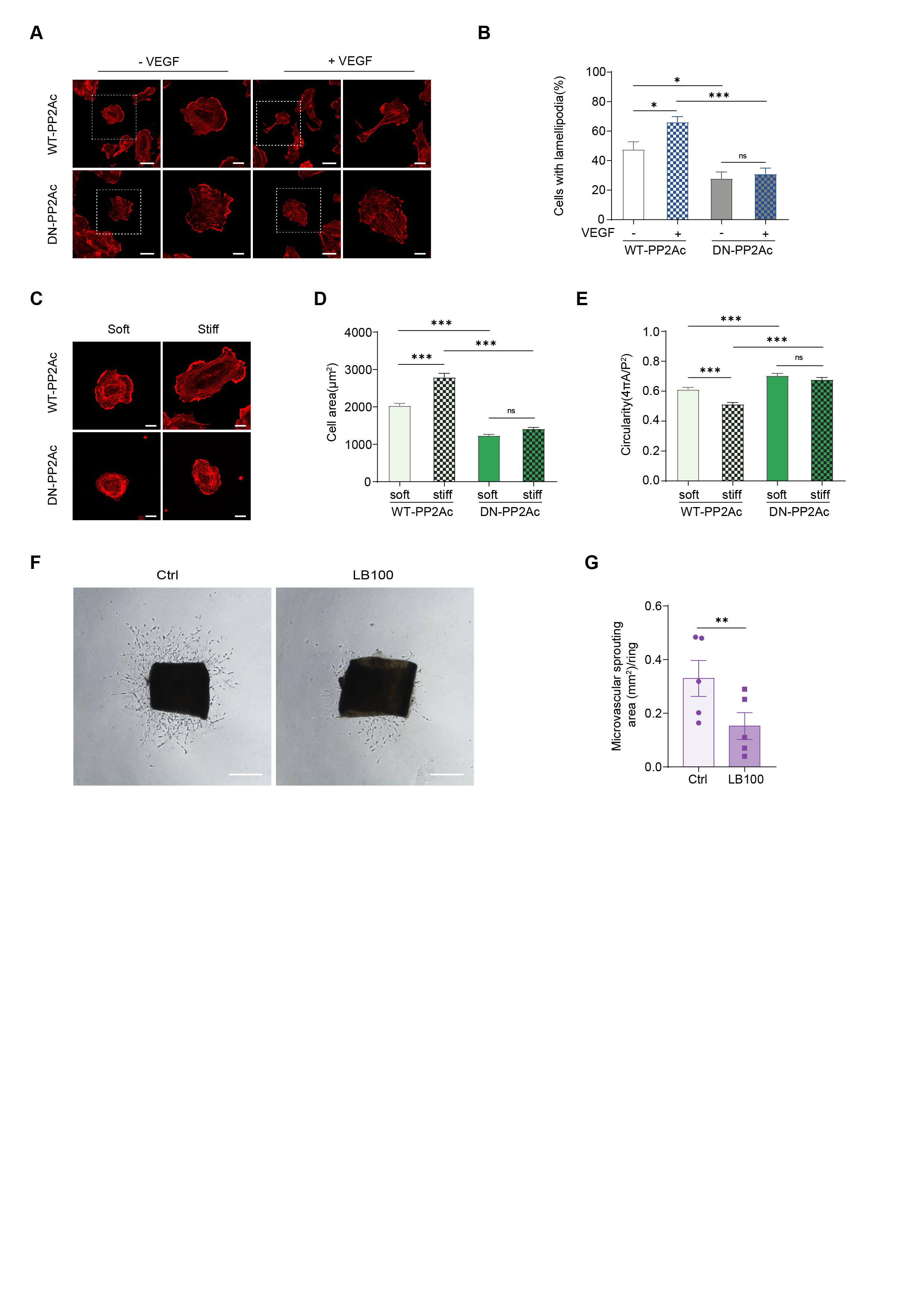

Supplement: Supplementary Figure 1 — PP2A activity is required for EC cytoskeleton change and sprouting. (A) Representative images of HUVECs stained with Phalloidin (actin) showing that lamellipodia formation upon VEGF (50 ng/mL for 30 min) treatment in WT-PP2Ac or DN-PP2Ac expressing HUVECs. (B) Quantification of the number of HUVECs with lamellipodia of (A). Cells in nine random fields of view (≈120 cells) were quantified. (C) Representative images of WT-PP2Ac or DN–PP2Ac expressing HUVECs seeded on soft (0.2 kpa) and stiff (20 kpa) hydrogels stained for Phalloidin (actin). (D,E) Quantification of individual cell area and circularity in (C). A, area; P, perimeter. Cells in 10 random fields of view (≈50 cells per condition) were quantified. (F) Representative images of mice aortic rings treated with vehicle (Ctrl) or LB100 (4 μM). (G) Quantification of microvascular sprouting area of (F). (n = 3–5 aortic ring per condition.) Data are shown as mean ± SEM, one-way ANOVA followed by Tukey’s multiple comparisons test in (B,D,E), two tailed Student’s t-test in G. ∗p < 0.05, ∗∗p < 0.01, and ∗∗∗p < 0.001, ns indicates not significant. Scale bars, 50 μm (lower magnification) and 20 μm (insets) in (A), 20 μm in (C), 500 μm in (F). [file Image_1.TIF]

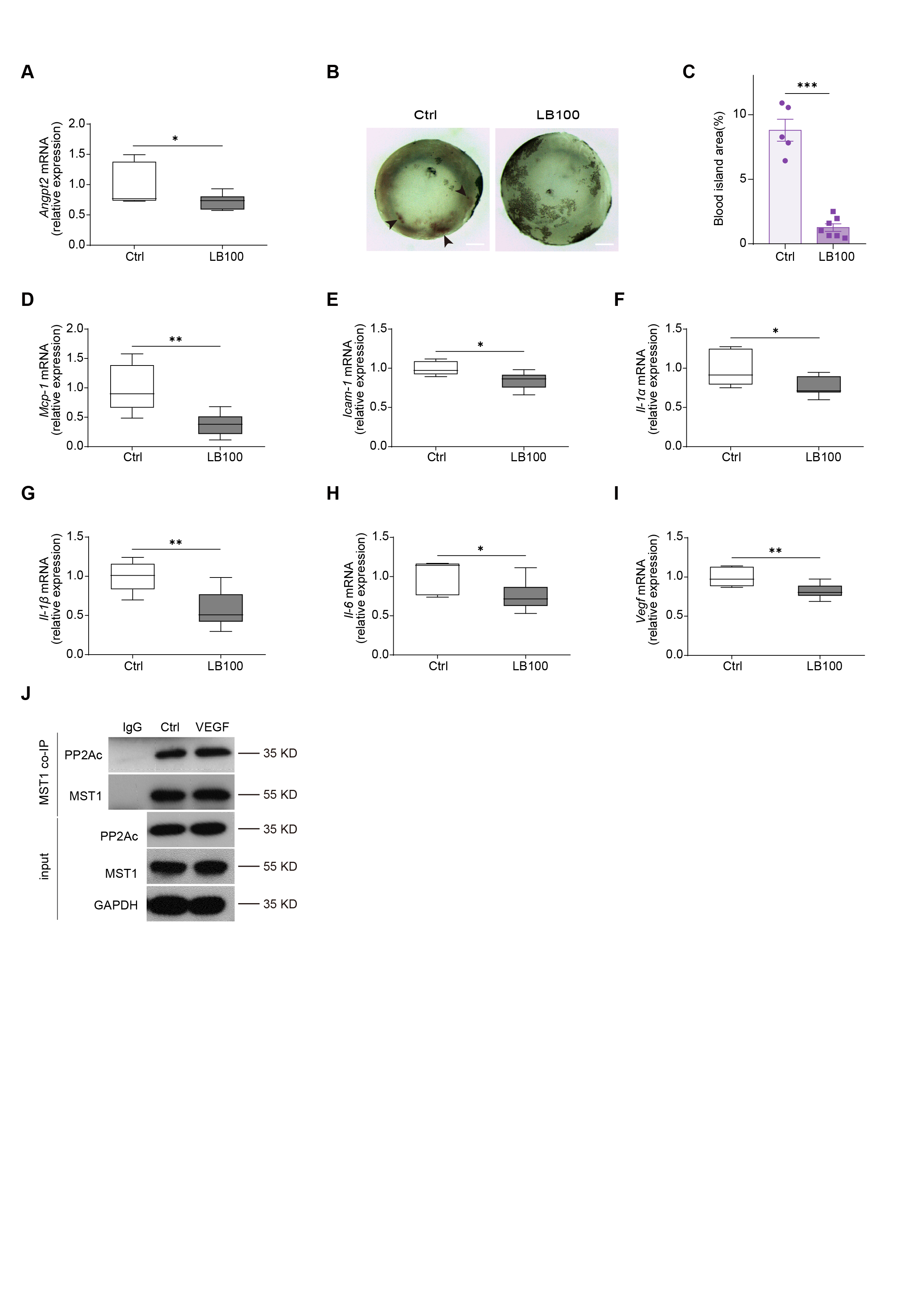

Supplement: Supplementary Figure 2 — PP2A inhibition reduces vascular leakage in the OIR model. (A) qPCR analysis of the expression of YAP target gene Angpt2 in retinas of vehicle or LB100 treated OIR pups. (n = 5 Vehicle and 9 LB100 mice per condition.) (B) Representative images of the outer surface of the OIR retinal cup at P17. Blood island formation indicates hemorrhages in the retinas. (C) Quantification of blood island area out of the total retinal area of (B). (n = 5 Vehicle and 7 LB100 mice per condition). (D–I) qPCR analysis of the expression of Mcp-1, Icam-1, Il-1a, Il-1b, Il-6, and Vegf in retinas treated as in (A). (n = 5 Vehicle and 9 LB100 mice per condition.) (J) Representative blots of the co-Immunoprecipitation (co-IP) of endogenous MST1 and PP2Ac in HUVECs stimulated with 50 ng/mL VEGF for 2 h. Data are shown as mean ± SEM, two tailed Student’s t-test. ∗p < 0.05, ∗∗p < 0.01, and ∗∗∗p < 0.001. Scale bars, 1 mm. [file Image_2.TIF]
